# Knowledge, Attitudes, and Practices on Urinary Schistosomiasis among Primary Schoolchildren in Nelson Mandela Bay, South Africa

**DOI:** 10.1155/2021/6774434

**Published:** 2021-11-02

**Authors:** Sydlynn Dorné Hambury, Anna D. Grobler, Paula Ezinne Melariri

**Affiliations:** ^1^Department of Environmental Health, Faculty of Health Sciences, Nelson Mandela University, Gqeberha (Port Elizabeth), South Africa; ^2^Department of Radiography, Faculty of Health Sciences, Nelson Mandela University, Gqeberha (Port Elizabeth), South Africa

## Abstract

**Background:**

Schistosomiasis remains a public health concern in South Africa (SA), with the highest prevalence of infection found among schoolchildren under the age of 15 years. Knowledge, attitude, and practices (KAP) studies on schistosomiasis among schoolchildren under the age of 15 years are lacking in the study area. The study therefore assessed primary schoolchildren in Grades 4–7 to determine their knowledge regarding schistosomiasis in the various ages represented in these grades.

**Methods:**

The study employed a quantitative descriptive, cross-sectional survey research design approach. A structured, close-ended, Likert-scale, self-administered questionnaire was used to collect data from 458 learners in Grades 4 to 7 aged from 9 to 16 years. Data were analysed using Statistica version 13 software. Bivariate and multivariate techniques were further used to analyse and describe the data and significant associations at *p* = 0.05 were further interrogated using Cohen's *d* and Cramér's *V*, to determine the practical significance.

**Results:**

Of the 458 learners who completed the questionnaire, 248 (54%) acknowledged having heard of schistosomiasis previously. There was a positive correlation between knowledge and attitude (0.779). The KAP scores were calculated as a percentage ranging between 0% and 100%, and this range was split into five equal width intervals 0–19%, 20–39%, 40–60%, 61–80%, and 81–100%. For knowledge, 210 (46%) of the participants obtained a score in the interval 0–19%. For attitudes, 237 (52%) of the participants obtained a score in the interval 0–19%. Therefore, the overall knowledge and attitudes among the study participants towards schistosomiasis were poor. There was a significant difference (*p* = 0.0005, *V* = 0.42 medium) between male and female participants relating to their practices. It was observed that a high percentage, 69 (15%) of males reported to swimming in slow-moving water compared to a significantly lower percentage, 9 (5%) of females. Furthermore, 23% of the participants reported that there was a river on the way to school.

**Conclusion:**

The study revealed that there was a positive correlation between knowledge and attitude. The overall knowledge and attitudes on schistosomiasis were poor. Furthermore, a gender-related difference based on practices emerged significant in the study. The findings are thus valuable in designing effective and targeted schistosomiasis control programmes.

## 1. Introduction

Schistosomiasis is prevalent in many developing countries, particularly in Africa. The disease is common in underprivileged communities with limited access to potable water and adequate sanitation [1]. Schistosomiasis affects nearly 240 million people worldwide, while another 700 million people are at risk of infection, especially children under the age of 15 years [1–4]. The disease is caused by trematodes of the genus *Schistosoma*. People become infected when they come into contact with cercariae-infested water bodies. It has been observed that children under the age of 15 years are more susceptible to infection due to certain play habits, such as fishing or swimming in infested water and lack of hygiene practices [1–4]. The disease causes morbidity in children below the ages of 15 years. If left untreated or undiagnosed, it can lead to a variety of long-term impacts such as anaemia and iron deficiency, leading to school absenteeism, poor academic performance, and increased high-school dropout rates [5].

Schistosomiasis can be controlled using key approaches adopted by the World Health Organization (WHO) which include adequate sanitation, potable water supply, effective treatment, and health education [1–4]. Health education programmes about schistosomiasis are necessary to influence a positive attitude and change the behaviour of people, especially children, in order to reduce and control the disease [6]. In addition, having adequate knowledge about the disease can also influence people's attitudes and behaviour leading to appropriate practices in controlling the transmission and spread of schistosomiasis [1–4]. Knowledge, attitude, and practices (KAP) studies are thus useful in investigating awareness levels about the disease among schoolchildren as well as determining risk factors for contracting the disease.

In South Africa (SA), the highest prevalence of infection is found among schoolchildren under the age of 15 years, with approximately 1 575 000 children infected [7]. In the Eastern Cape (EC) province of SA, where cases of schistosomiasis have been reported, the current KAP of schoolchildren in the study area (KwaNobuhle) is unknown. Several studies suggest that knowledge influences attitude and eventually the practices of children [8]. The dearth of information on the KAP of schoolchildren in the study area makes it challenging to design effective and targeted control measures.

## 2. Research Methods and Design

### 2.1. Study Area

**Figure 1:**
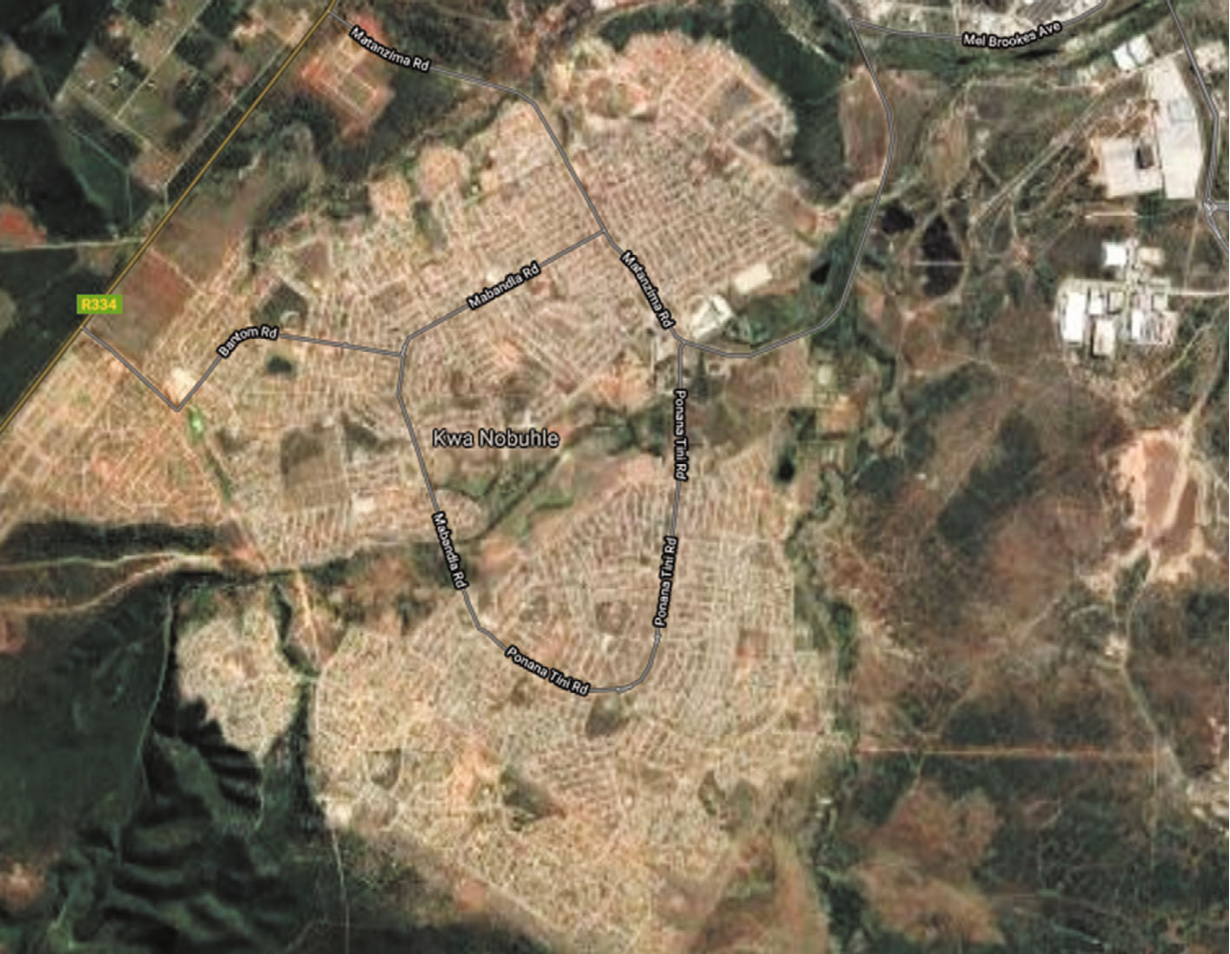
A map depicting the study area.

The study was conducted in KwaNobuhle, a large township (suburb) situated on the outer edge of Kariega (Uitenhage) within the Nelson Mandela Bay, EC province of SA. The total area of KwaNobuhle is approximately 23.48 km^2^ with geographical coordinates of 33.8118°S, 25.3845°E. KwaNobuhle has a total population of approximately 107 474 people according to a census done in the year 2011. The majority of people from KwaNobuhle work in the manufacturing and trade industry [9]. The area is characterised by short and warm summers from December to March, with temperatures above 27°C. The winters are cool, dry, and windy from May to September, with daily temperatures below 22°C. Kwanobuhle receives about 331 mm of rain per year, with rainfall occurring throughout the year. The lowest rainfall (3.4 mm) occurs in August and the highest (50 mm) in April. The area is characterised by streams, ponds, plants and fauna, and overgrown vegetation that constitute the hydrologic and geographic features [10]. These stagnant water bodies in the study area serve as potential habitats to the disease intermediate host. A topographical view of the study area is depicted in Figure 1.

### 2.2. Ethical Considerations

Before data collection, the study received ethical approval from the Research Ethics Committee: Human (REC-H) of the Nelson Mandela University (H18-HEA-ENV-003) and gatekeepers permissions were obtained from the Eastern Cape Department of Education (ECDOE) and the principal of the four selected primary schools. Schoolchildren who were requested to participate in the study were informed about the research and assented to participate before participation. Since participants were under the age of 18 years, consent of parent(s) or legal guardian(s) was sought prior to their recruitment. Further precautions were also taken not to link participants or the selected schools to the findings of the study.

### 2.3. Study Design

The study employed a quantitative descriptive, cross-sectional survey that elicited responses on the knowledge, attitudes, and practices on schistosomiasis among schoolchildren in Grades 4 to 7 from selected primary schools in KwaNobuhle. The study was conducted in October 2019.

### 2.4. Study Population and Sampling Strategy

The study population comprised Grade 4 to 7 learners aged from 9 to 16 years from four selected primary schools in KwaNobuhle. The learners in Grades 4 to 7 from the four selected primary schools in the study area are under the age of 15 years. However, there are some learners who repeated a school year. Consequently, these learners within the specific grades (Grades 4 to 7) included in the study may be up to the age of 16 years. The total population from the four selected primary schools was 1506 learners, of which 797 (53%) were males and 709 (47%) were females. The four primary schools in KwaNobuhle were purposively selected to be included in the study due to the geographical location of the schools being close to isolated stagnant water bodies. The sample size required for this study was calculated using the Yamane's formula for determining the sample size in a survey research [11]:

(1) $$\begin{array}{c} n=\frac{N}{1}+{Ne}^{2}, \end{array}$$

where *n* is the sample size, *N* is the population (of the proposed study group), and *e* is the margin of error (at 95% confidence level).

For this study with a population of 1506 and 5% margin of error, the formula equates to a minimum sample size of

(2) $$\begin{array}{c} n=\frac{1506}{1}+1506x{\left(0.05\right)}^{2}, \end{array}$$

317 is a proportion of 0.21 (a percentage of 21%) of the population *N*.

A stratified cluster-sampling method was used to select the study participants because the total population from each school was divided into different grades (Grades 4 to 7), and then the classes were randomly selected from each grade per school. This method allowed a predetermined number of children from each class per grade to be included in the study. This ensured that each child had an equal opportunity of being selected from the population to be included in the study. This method also ensured that the sample proportions regarding gender and grade were equivalent to the corresponding population proportions. Participants that were eligible for inclusion were selected based on the following criteria: (1) children in Grades 4 to 7 from the four selected primary schools in KwaNobuhle, (2) children present on the day of the survey for the study, and (3) children who had assented to participate and had received consent from their parents(s) or legal guardian(s). For a representative sample size, a minimum total of 317 participants was required, but a total of 458 learners participated in the study based on the required sample size and inclusion/exclusion criteria.

### 2.5. Data Collection

**Figure 2:**
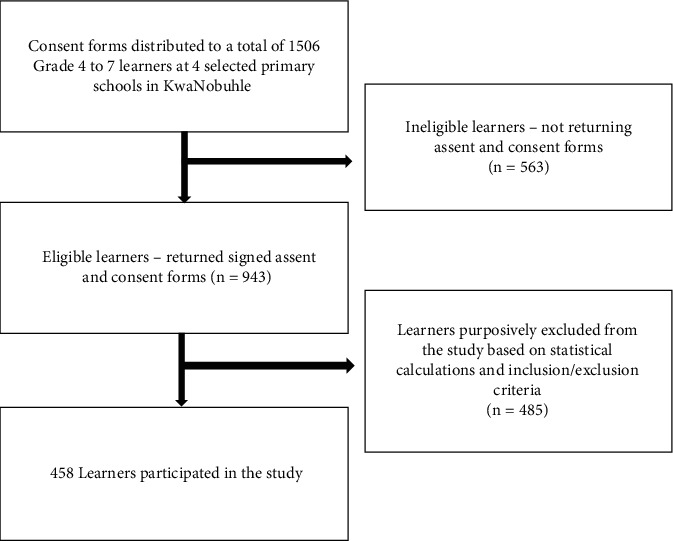
Flow chart of the participation and compliance in the present study.

A structured, closed-ended, Likert-scale, self-administered questionnaire was used to collect data from 458 learners who participated in the study (Figure 2). The questionnaire was back-translated to ensure that the original English text had been properly translated into isiXhosa. The questionnaire had six sections: demographic questions, environmental factor questions, knowledge, attitudes, practices, and health-related symptom questions about schistosomiasis. A statistician was consulted to ensure that the scores derived from the collected data accurately measured respondents' scores for the knowledge, attitudes, practice, and health (KAPH) factors. The pilot study conducted also ensured the validity of the research instrument.

### 2.6. Data Analyses

The information obtained from the Likert-scale questionnaire was captured and analysed using Microsoft Office 365 pro-plus 2019 version and Statistica version 13 software. The data processing included checking of the data for errors and the calculation of summated scores for the knowledge, attitudes, practice, and health (KAPH) items in the questionnaire. The scores for the KAPH factors were calculated to range between 0 and 100 points. The formula to calculate a respondent's score for factor *F* was *SF* = (100 × *AF*)/*MF*, where *AF* is the aggregated sum of the points awarded for the respondent's responses and *M*_*F*_ is the maximum number of points that can be achieved for factor *F*.

Descriptive and inferential statistics were used to analyse and describe the data and included tests such as *t*-tests, Analysis of Variance (ANOVA), and chi-square (*χ*^2^) tests. The Pearson product-moment correlations were calculated to investigate the relationships among the KAPH factors. Hypothesis testing was conducted with a significance level of *α* = 0.05; significant associations were further interrogated using Cohen's *d*, for tests involving sample means, and Cramér's *V*, for tests involving frequencies, to determine the practical significance. Cohen's values larger than 0.20 and Cramér's *V* larger than 0.10 indicate practically significant results.

## 3. Results

### 3.1. Demographic and Associated Environmental Factors among Participants

**Figure 3:**
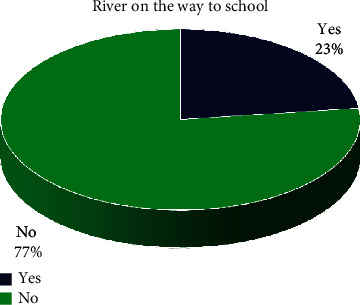
River on the way to school.

A total of 458 participants comprising 250 (55%) females and 207 (45%) males participated in the study. The majority, 238 (53%) of the participants were between the ages of 12 and 13 years (Table 1). Most of the participants, 132 (29%) were in Grade 6. The findings from the study revealed that a few, 20 (15%) participants who lived near slow-moving water visited the water regularly, often, or weekly compared to a significantly (*p* = 0.003, *V* = 0.16 small) lower percentage (7%) of participants that visited the water regularly, often, or weekly and did not live near slow-moving water (Table 1). Regarding water bodies being in close proximity to living areas, 23% participants reported that there was a river on the way to school (Figure 3).

### 3.2. Schistosomiasis Awareness and Knowledge among Participants

**Figure 4:**
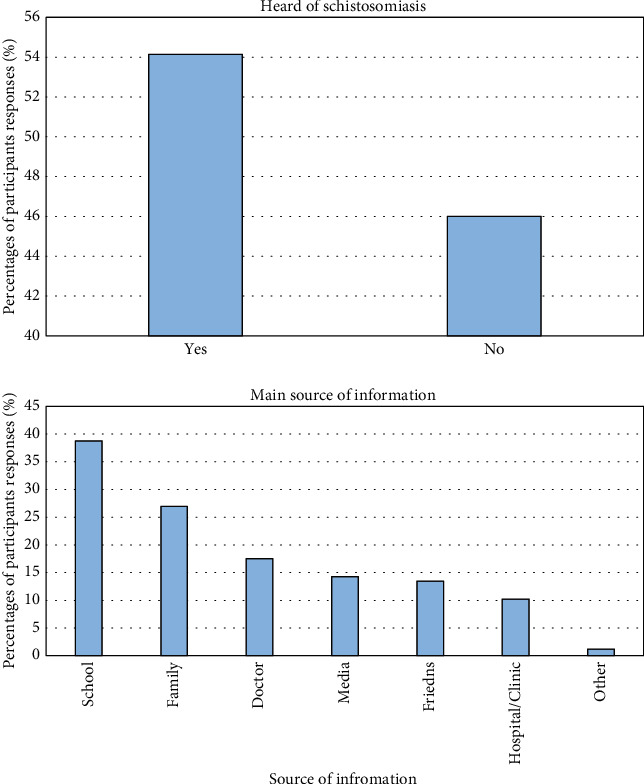
Study participants' awareness of schistosomiasis and main source of information.

**Figure 5:**
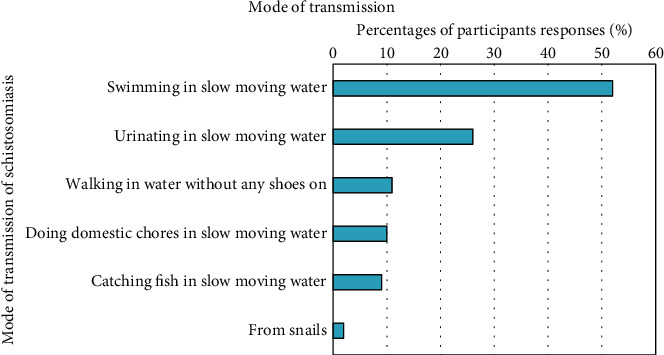
Knowledge about the mode of transmission of schistosomiasis among study participants.

**Figure 6:**
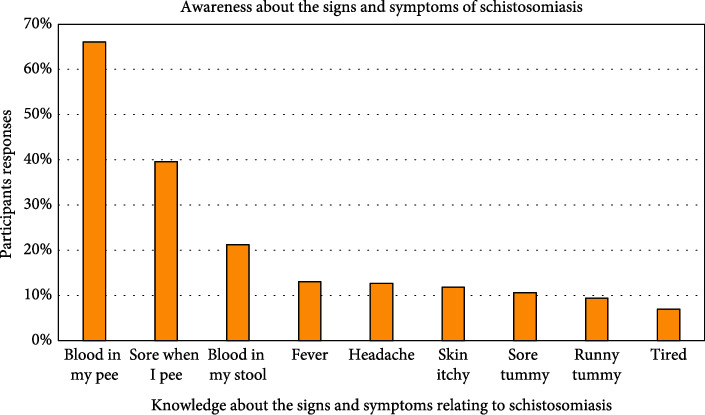
Knowledge about the signs and symptoms of schistosomiasis among study participants.

Of the 458 participants who enrolled for the current study, 248 (54%) acknowledged having heard of Bilharzia (schistosomiasis) previously, with schools, 95 (38%) being the main source of information (Figure 4). Almost half, 128 (52%) of the participants knew that swimming in slow-moving water might lead to the cause of the disease, while, 64 (26%) also correctly answered that urinating in the water might lead to contracting schistosomiasis (Figure 5). In terms of knowledge about the signs and symptoms relating to schistosomiasis, most of the participants (162, 65%) knew that the disease caused blood in urine, 97 (39%) painful urination, 52 (21%) blood in stool, and 26 (10%) abdominal pains (Figure 6). The scores for knowledge were calculated as a percentage ranging between 0% and 100%. This range was split into five equal-width intervals 0–19%, 20–39%, 40–60%, 61–80%, and 81–100%. For knowledge, 210 (46%) of the participants obtained a score in the interval 0–19%. Therefore, the overall awareness about schistosomiasis among these participants was low (Table 2).

### 3.3. Attitudes towards Schistosomiasis among Participants

**Figure 7:**
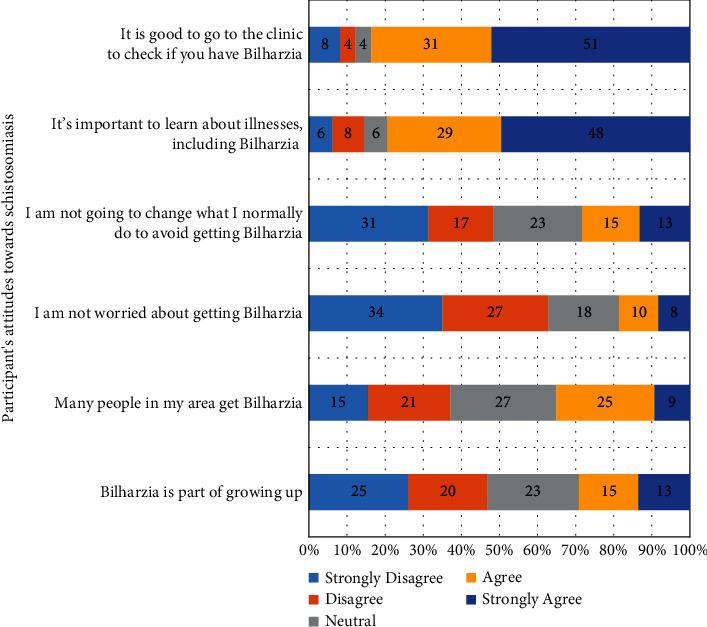
Study participants' attitudes towards schistosomiasis.

Of the participants who had prior knowledge concerning schistosomiasis, the majority, 127 (51%) strongly agreed that it was good to go to the clinic to check if one had schistosomiasis; and 120 (48%) strongly agreed that it was important to learn about illnesses, including schistosomiasis; and 31 (13%) strongly agreed that they were not going to change what they normally did to avoid getting schistosomiasis (Figure 7). The scores for attitudes were calculated as a percentage ranging between 0% and 100%. This range was split into five equal-width intervals 0–19%, 20–39%, 40–60%, 61–80%, and 81–100. For attitudes, 237 (52%) of the participants obtained a score in the interval 0–19%. Therefore, the overall attitudes towards schistosomiasis among these participants were low, based on the attitude scores (Table 3).

### 3.4. Practices Relating to Schistosomiasis among Participants

The results relating to the water-related practices among the 458 study participants showed that 79 (17%) swam in slow-moving water, 40 (9%) urinated in the water, and 36 (8%) walked in the water. The findings from the current study revealed that there was a significant difference between male and female participants relating to their practices. It was observed that a high percentage, 69 (15%) of males reported to swimming in slow-moving water compared to a significantly (*p* < 0.0005, *V* = 0.42 medium) lower percentage, 9 (2%) of females that did. Likewise, the results revealed a significantly (*p* = 0.001, *V* = 0.28 small) higher percentage, 68 (15%) of males that urinated in slow-moving water compared to that of females 40 (9%) (Table 4). The scores for practices were calculated as a percentage ranging between 0% and 100%. This range was split into five equal-width intervals 0–19%, 20–39%, 40–60%, 61–80%, and 81–100%. For practices, 264 (58%) of the participants obtained a score in the interval 61–80%. Therefore, the participants' overall practices were fairly good (Table 5).

### 3.5. Haematuria (Blood in Urine) among Participants

Out of the 458 study participants, 52 (11%) had gone to the clinic to be checked for schistosomiasis. Interestingly, 29 (6%) reported haematuria (blood in urine) and 429 (94%) did not observe haematuria. Furthermore, 54 (12%) experienced no symptoms and 18 (4%) had had blood in their stool (Table 6).

### 3.6. Multivariate Analysis of the KAPH Factors Relating to Schistosomiasis and the Demographic Variables among Participants

A chi-square test was performed to determine the association between variables. Associations between variables were considered significant at a 95% confidence interval with *p* < 0.05. The results of the multivariate analysis of the KAPH factors relating to schistosomiasis among the study participants showed that there was a positive correlation (0.779) between knowledge and attitude (Table 7). This suggests that participants with higher levels of knowledge typically have a more positive attitude and that those with low levels of knowledge usually have a negative attitude. Analysis of knowledge relative to age using ANOVA indicated that there was no significant difference between the knowledge means of the three age groups being compared (*p* = 0.100). The mean knowledge level of participants aged from 14 to 16 years (*M* = 25.98) was lower than that of those participants aged from 9 to 11 years (*M* = 32.39) as well as those aged from 12 to 13 years (*M* = 29.58). The mean knowledge level of participants aged from 14 to 16 years (*M* = 25.98) was lower than that of those participants aged from 9 to 11 years (*M* = 32.39) as well as that of those aged from 12 to 13 years (*M* = 29.58). The Odds Ratio (OR) for knowledge relative to age was 0.80 (95% CI: 0.54; 0.18). With regard to knowledge by grade, the analysis of knowledge per grade using ANOVA and inferential statistics (Scheffé test and Cohen's d) indicated that there was a significant difference between the knowledge means of Grade 5 and Grade 6 compared to those of Grade 4 and Grade 7 (*p* = 0.001). The mean knowledge level of Grade 5 (*M* = 36.04) and Grade 6 (*M* = 31.12) participants was significantly higher than that of those participants in Grade 4 (*M* = 27.80) and Grade 7 (*M* = 26.02). The OR for knowledge per grade was 0.79 (95% CI: 0.55; 1.14). The poor level of knowledge among the Grade 7 participants compared with the lower grades could be due to interest and attitudes towards schistosomiasis. With regard to attitudes by grade, the current study observed that the lowest mean values among the four grades were recorded in Grade 7 with a mean value of 18.57. The OR for attitude per grade was 0.73 (95% CI: 0.50; 1.06). Participants in Grades 4, 5, and 6 had a higher level of attitudes, with a mean value of 21.75, 27.28, and 27.26, respectively. With regard to practice by grade, it was observed that participants in Grades 4, 5, 6, and 7 had fair mean scores, with mean values of 67.91, 69.84, 67.73, and 69.43, respectively. Regarding the responses on health-related symptoms of schistosomiasis among participants relative to age, the ANOVA result indicated that there was no statistically significant difference between the mean values of the health of the three age groups being compared (*p* = 0.240). It was observed that the mean values of participants aged from 14 to 16 years (*M* = 70.24) were higher compared to the mean values of participants aged from 9 to 11 years (*M* = 64.51) and 12 to 13 years (*M* = 65.80). The responses on health-related symptoms of schistosomiasis among the participants in the three age groups showed no relationship between practice and health.

## 4. Discussion

The study revealed that there were some environmental risk factors that increased the possibility for individuals to contract schistosomiasiswhen in contact with infested water bodies. Such factors include living close to water bodies and having rivers on the way to school. The findings from the study further revealed that a few, 20 (15%) participants who lived near slow-moving water visited the water regularly, often, or weekly compared to a significantly (*p* = 0.003, *V* = 0.16 small) lower percentage, 21 (7%) of participants that visited the water regularly, often, or weekly and did not live near slow-moving water. This concurs well with a study conducted in Northern Senegal, which reported that children living in areas where the river was more accessible had higher levels of schistosomiasis infection than children who could not readily access rivers that easily [12]. Furthermore, a few participants (23%) reported that there was a river on the way to school which was also a significant risk factor for contracting schistosomiasis. A previous study reported that children attending school situated close to rivers or dams had a higher rate of schistosomiasis infections than those attending school situated further away [13].

The research findings indicated that the overall knowledge and attitudes towards schistosomiasis among the participants were low. Previous studies have reported that lower levels of knowledge in relation to schistosomiasis often lead to poor prevention practices [14]; therefore, knowledge about the disease is the key as adequate knowledge may lead to behavioural change, like avoiding contact with infested waters. The study further showed that the overall practices of the majority of the participants were reasonably good. However, there was a significant difference between male and female participants relating to their practices. It was observed that a high percentage, 69 (15%) of males reported to swimming in slow-moving water compared to a significantly (*p* < 0.0005, *V* = 0.42 medium) lower percentage, 9 (2%) of females that did. A previous study conducted in Northern Senegal reported that the riskiest practices were related to water contact behaviour. Boys play in the river more than girls do, confirming previous reports that playing in stagnant or slow-moving water bodies is associated with schistosomiasis infection [12]. In view of the participants' responses towards health-related symptoms of schistosomiasis, the findings showed that only a few, 29 (6%) participants reported experiencing haematuria (blood in urine).

The multivariate analysis of the KAPH factors relating to schistosomiasis among the study participants showed that there was a positive correlation between knowledge and attitude. The study further revealed that there was a decline in the level of knowledge among the participants in the older age group compared to that of the younger age group. The knowledge level of participants aged from 14 to 16 years was lower than the knowledge level of participants aged from 9 to 11 years and from 12 to 13 years. Participants aged 9 to 11 years displayed better attitudes than the older group of participants aged 14 to 16 years. The findings from the study further revealed that participants with lower knowledge displayed a more negative attitude than those with a higher level of knowledge and who displayed a better attitude. Previous studies suggest that there is a link between attitudes and knowledge. Liking a course or displaying positive attitudes towards it will, in turn, develop positive learning. Attitudes are also an important factor that increases the quality of education [15]. Furthermore, previous studies claim that low levels of attitude towards problem solving or a particular topic are displayed as the grade raises [15].

Owing to the limited number of similar studies conducted within the EC province of SA, further comparisons of the study findings with other regions within the EC province were therefore not possible. It is suggested that this study be replicated at schools all over the EC province. Further studies are thus needed to determine the level of knowledge about schistosomiasis among the Grade 7 participants compared to the level of knowledge displayed among participants in the lower grades. There is also a need for regular assessment of the prevalence of urinary schistosomiasis among schoolchildren in the study area, EC province, and other parts of the country to determine the current prevalence, disease incidence, and intensity of infection.

## 5. Conclusion

The overall knowledge and attitudes towards schistosomiasis among the participants were poor. Health education programmes and mass treatment at school, putting up notice boards at the banks of stagnant-water bodies, controlling fresh water snails, building of public swimming pools, and improving sanitation are all key measures for controlling the disease. Further studies are thus needed to determine the factors associated with low level of knowledge about schistosomiasis among the Grade 7 learners compared to the high level of knowledge displayed among learners in the lower grades. There is an acute need for regular assessment of the prevalence of urinary schistosomiasis among schoolchildren in the study area, EC province, and other parts of the country to determine the current prevalence, disease incidence, and intensity of infection. This will be valuable in determining the status of schistosomiasis of a wider population so that preventative and control measures can be implemented.

## Figures and Tables

**(a):** 

Variable	No of respondents (*n*)	Percentage (%)
Gender		
Male	207	45
Female	250	55
Age (years)		
9–11	166	36
12–13	238	53
14–16	49	11
Grade		
4	92	20
5	111	24
6	132	29
7	123	27
School		
A	91	20
B	133	29
C	150	33
D	84	18
Toilet at home		
Yes	449	98
No	9	2
Where is the toilet		
Inside	302	68
Outside	144	32
Slow-moving water near house		
Yes	136	30
No	322	70

**(b):** 

Participants regularity of visiting slow-moving water			
Slow moving water near house	Never *n* (%)	Seldom *n* (%)	Regularly/often/weekly *n* (%)
Yes	73 (54)	41 (31)	20 (15)
No	213 (70)	72 (24)	21 (7)
Chi-square (*χ*^2^)			
*p* value	0.003		
*v* value	0.16 small		

**Table 2:** Knowledge scores among study participants.

Variable	Intervals expressed as a percentage (%)				
Knowledge	Lowest 0 to 19	Lower 20 to 39	Middle 40 to 60	Higher 61 to 80	Highest 81 to 100
Participants *n* (%)	210 (46%)	86 (19%)	134 (29%)	28 (6%)	0 (0%)

**Table 3:** Attitude scores among study participants.

Variable	Intervals expressed as a percentage (%)				
Attitude	Lowest 0 to 19	Lower 20 to 39	Middle 40 to 60	Higher 61 to 80	Highest 81 to 100
Participants *n* (%)	237 (52%)	66 (14%)	107 (23%)	42 (9%)	6 (1%)

**Table 4:** Water practices among study participants (*n* = 458).

Variable	Water practices, *n* (%)									
	Swim	Walk in water	Catch fish	Wash clothes	Wash dishes	Wash myself	Get water to drink	Get water for washing	Throw tins, paper, or bottles into the water	Urinate in water
Participants	79 (17)	36 (8)	23 (4)	16 (3)	18 (4)	27 (6)	16 (3)	15 (3)	17 (4)	40 (9)
Age (years)										
9-11	22 (5)	9 (2)	4 (1)	6 (1)	6 (1)	12 (3)	5 (1)	5 (1)	4 (1)	31 (7)
12-13	45 (10)	26 (6)	18 (4)	10 (2)	11 (2)	14 (3)	11 (2)	9 (2)	12 (3)	69 (15)
14-16	11 (2)	1 (0)	1 (0)	0 (0)	1 (0)	1 (0)	0 (0)	1 (0)	1 (0)	6 (1)
*χ* ^2^										
*p* value	0.127	0.068	0.081	0.365	0.792	0.240	0.325	0.900	0.484	0.028
*v* value	—	—	—	—	—	—	—	—	—	0.22 small
Gender										
Female	9 (2)	10 (2)	3 (1)	10 (2)	7 (2)	13 (3)	7 (2)	5 (1)	12 (3)	40 (9)
Male	69 (15)	26 (6)	20 (4)	6 (1)	11 (2)	14 (3)	9 (2)	10 (2)	5 (1)	68 (15)
*χ* ^2^										
*p* value	<0.0005	0.903	0.071	0.001	0.299	0.012	0.153	0.666	<0.0005	0.001
*v* value	0.42 medium	—	—	0.27 small	—	0.21 small	—	—	0.64 medium	0.28 small
Grade										
4	14 (3)	7 (2)	2 (0)	3 (1)	4 (1)	8 (2)	4 (1)	4 (1)	2 (0)	19 (4)
5	12 (3)	7 (2)	3 (1)	4 (1)	3 (1)	5 (1)	2 (0)	1 (0)	3 (1)	20 (4)
6	25 (5)	16 (3)	10 (2)	2 (0)	5 (1)	6 (1)	5 (1)	4 (1)	7 (2)	43 (9)
7	28 (6)	6 (1)	8 (2)	7 (2)	6 (1)	8 (2)	5 (1)	6 (1)	5 (1)	26 (6)
*χ* ^2^										
*p* value	0.026	0.344	0.287	0.196	0.801	0.272	0.732	0.286	0.813	0.447
*v* value	0.26 small	—	—	—	—	—	—	—	—	—

**Table 5:** Water practice scores among study participants.

Variable	Intervals expressed as a percentage (%)				
Water practices	Lowest 0 to 19	Lower 20 to 39	Middle 40 to 60	Higher 61 to 80	Highest 81 to 100
Participants *n* (%)	3 (1%)	34 (7%)	127 (28%)	264 (58%)	30 (7%)

**Table 6:** Health-related symptoms relating to schistosomiasis among study participants (*n* = 458).

Variable	Health-related symptoms, *n* (%)									
	Gone to the clinic to check for bilharzia	Cough	Skin itchy	Headache	Fever	Sore tummy	Runny tummy	Blood in my pee (urine)	Blood in my stool	No symptoms
Participants	52 (11)	274 (60)	107 (23)	176 (38)	154 (34)	138 (30)	113 (25)	29 (6)	18 (4)	54 (12)
Age (years)										
9-11	18 (4)	107 (23)	35 (8)	65 (14)	56 (12)	54 (12)	46 (10)	10 (2)	10 (2)	17 (4)
12-13	22 (5)	140 (31)	62 (14)	98 (21)	84 (18)	67 (15)	59 (13)	12 (3)	6 (1)	31 (7)
14-16	10 (2)	26 (6)	8 (2)	13 (3)	10 (2)	16 (3)	8 (2)	6 (1)	2 (0)	6 (1)
*χ* ^2^										
*p* value	0.003	0.288	0.246	0.159	0.128	0.594	0.269	0.161	0.207	0.695
*v* value	0.18 small	—	—	—	—	—	—	—	—	
Gender										
Female	14 (3)	155 (34)	60 (13)	109 (24)	86 (19)	76 (17)	55 (12)	10 (2)	8 (2)	33
Male	38 (8)	119 (26)	46 (10)	67 (15)	67 (15)	62 (14)	58 (13)	19 (4)	9 (2)	21
*χ* ^2^										
*p* value	<0.0005	0.327	0.654	0.014	0.647	0.917	0.138	0.024	0.519	0.314
*v* value	0.27 small	—	—	0.11 small	—	—	—	0.11 small	—	—
Grade										
4	11 (2)	61 (13)	24 (5)	38 (8)	30 (7)	33 (7)	25 (5)	8 (2)	9 (2)	14 (3)
5	18 (4)	61 (13)	20 (4)	39 (9)	27 (6)	28 (6)	32 (7)	7 (2)	3 (1)	5 (1)
6	13 (3)	61 (13)	30 (7)	59 (13)	47 (10)	40 (9)	31 (7)	11 (2)	5 (1)	16 (3)
7	10 (2)	57 (12)	32 (7)	40 (9)	50 (11)	37 (8)	25 (5)	3 (1)	1 (0)	19 (4)
*χ* ^2^										
*p* value	0.099	0.895	0.387	0.184	0.063	0.439	0.442	0.179	0.016	0.040
*v* value	—	—	—	—	—	—	—	—	0.15 small	0.13 small

**Table 7:** Multivariate analysis of knowledge, attitudes, practices, and health factors relating to schistosomiasis among the study participants (*n* = 458).

Variable	Chi-square (*χ*^2^), *n* (%)		*p* value	Correlations	Odds Ratio (95% Cl)
	Lower (<Med.) *n* (%)	Higher (>Med.) *n* (%)			
Knowledge				0.779	
Age (years)			0.100		0.80 (0.54; 0.18)
9-11	71 (43)	95 (57)			
12-13	115 (48)	123 (52)			
14-16	30 (61)	19 (39)			
Gender			0.218		1.21 (0.83; 1.75)
Female	124 (50)	126 (50)			
Male	93 (45)	114 (55)			
Grade			0.001		0.79 (0.55; 1.14)
4	49 (53)	43 (47)			
5	41 (37)	70 (63)			
6	55 (42)	77 (58)			
7	73 (59)	50 (41)			
Attitudes				0.779	
Age (years)			0.016		0.64 (0.44; 0.95)
9-11	68 (41)	98 (59)			
12-13	120 (50)	118 (50)			
14-16	32 (65)	17 (35)			
Gender			0.458		1.09 (0.76; 1.58)
Female	124 (50)	126 (50)			
Male	98 (47)	109 (53)			
Grade			0.020		0.73 (0.50; 1.06)
4	49 (53)	43 (47)			
5	41 (37)	70 (63)			
6	57 (43)	75 (57)			
7	76 (62)	47 (38)			
Practices				—	
Age (years)			0.221		0.94 (0.63; 1.40)
9-11	61 (37)	105 (63)			
12-13	95 (40)	143 (60)			
14-16	14 (29)	35 (71)			
Gender			*p* < 0.0005		0.25 (0.17; 0.38)
Female	58 (23)	192 (77)			
Male	113 (55)	94 (45)			
Grade			0.791		1.03 (0.70; 1.51)
4	36 (39)	56 (61)			
5	41 (37)	70 (63)			
6	55 (42)	77 (58)			
7	40 (33)	83 (67)			
Responses on schistosomiasis health-related symptoms				—	
Age (years)			0.240		1.72 (1.13; 2.61)
9-11	66 (40)	100 (60)			
12-13	88 (37)	150 (63)			
14-16	13 (27)	36 (73)			
Gender			0.798		1.06 (0.73; 1.56)
Female	93 (37)	157 (63)			
Male	74 (36)	133 (64)			
Grade			0.473		0.88 (0.60; 1.29)
4	41 (45)	51 (55)			
5	30 (27)	81 (73)			
6	53 (40)	79 (60)			
7	44 (36)	79 (64)			

## Data Availability

The data that support the findings of this study are available from the corresponding author (Sydlynn Dorné Hambury) upon reasonable request.
